# Genome-wide analysis of changes in miRNA and target gene expression reveals key roles in heterosis for Chinese cabbage biomass

**DOI:** 10.1038/s41438-021-00474-6

**Published:** 2021-03-01

**Authors:** Peirong Li, Tongbing Su, Deshuang Zhang, Weihong Wang, Xiaoyun Xin, Yangjun Yu, Xiuyun Zhao, Shuancang Yu, Fenglan Zhang

**Affiliations:** 1 Beijing Vegetable Research Center (BVRC), Beijing Academy of Agriculture and Forestry Sciences (BAAFS), Beijing, 100097 China; 2 Key Laboratory of Biology and Genetic Improvement of Horticultural Crops (North China), Ministry of Agriculture, Beijing, 100097 China; 3 Beijing Key Laboratory of Vegetable Germplasm Improvement, Beijing, 100097 China

**Keywords:** Non-coding RNAs, Plant breeding, Transcriptomics

## Abstract

Heterosis is a complex phenomenon in which hybrids show better phenotypic characteristics than their parents do. Chinese cabbage (*Brassica rapa* L. spp. *pekinensis*) is a popular leafy crop species, hybrids of which are widely used in commercial production; however, the molecular basis of heterosis for biomass of Chinese cabbage is poorly understood. We characterized heterosis in a Chinese cabbage F_1_ hybrid cultivar and its parental lines from the seedling stage to the heading stage; marked heterosis of leaf weight and biomass yield were observed. Small RNA sequencing revealed 63 and 50 differentially expressed microRNAs (DEMs) at the seedling and early-heading stages, respectively. The expression levels of the majority of miRNA clusters in the F_1_ hybrid were lower than the mid-parent values (MPVs). Using degradome sequencing, we identified 1,819 miRNA target genes. Gene ontology (GO) analyses demonstrated that the target genes of the MPV-DEMs and low parental expression level dominance (ELD) miRNAs were significantly enriched in leaf morphogenesis, leaf development, and leaf shaping. Transcriptome analysis revealed that the expression levels of photosynthesis and chlorophyll synthesis-related MPV-DEGs (differentially expressed genes) were significantly different in the F_1_ hybrid compared to the parental lines, resulting in increased photosynthesis capacity and chlorophyll content in the former. Furthermore, expression of genes known to regulate leaf development was also observed at the seedling stage. Arabidopsis plants overexpressing *BrGRF4.2* and bra-miR396 presented increased and decreased leaf sizes, respectively. These results provide new insight into the regulation of target genes and miRNA expression patterns in leaf size and heterosis for biomass of *B. rapa*.

## Introduction

Heterosis is a biological phenomenon in which hybrids have better phenotypic characteristics than their parents do for traits such as biomass production, grain yield, growth rate, and stress resistance^1–3^. Many heterotic crops, such as those of hybrid rice, maize, and many vegetable species, have been developed extensively worldwide. To date, dominance, overdominance, epistasis, and several other theories have been proposed to explain heterosis from multiple genetic perspectives^4–6^. However, these genetic models have not adequately described the molecular basis of heterosis^7,8^, and why hybrids display superior growth and fertility is still a mystery that awaits further exploration^9^.

Plant microRNAs (miRNAs) regulate gene expression through epigenetic regulation and posttranscriptional mechanisms^10^. Most miRNAs cause target gene degradation through the RNA-induced silencing complex effector^11^. Indeed, expression differences of miRNAs have been identified in many hybrid crops compared with their parental lines^1^. A recent study showed that the expression level of most miRNA clusters in F_1_ hybrids of *Brassica napus* was higher than that of their parents^12^. In both Arabidopsis and hexaploid wheat hybrids, changes in miRNA expression result in nonadditive expression of target genes, thereby affecting growth, vitality and adaptability^13,14^. Several miRNAs and their target genes have thus far been found to contribute to leaf development in various plant species, including miR156-*SQUAMOSA PROMOTER BINDING PROTEIN-LIKE* (*SPL*) in Arabidopsis^15^, miR160-*AUXIN RESPONSE FACTOR* (*ARF*) in tomato^16^, miR319-*TEOSINTE BRANCHED*/*CYCLOIDEA*/*PROLIFERATING CELL FACTOR* (*TCP*)^17^, and miR396-*GROWTH REGULATING FACTOR* (*GRF*)^18^. Nevertheless, the miRNA-pathway networks of leaf development, which contribute to the increased leaf biomass of hybrids, should be further analyzed.

Biomass heterosis is a distinct phenotype in which the production of larger leaves of hybrids is due to the increase in cell number and/or cell size^19^. For example, leaf heterosis in Arabidopsis hybrids is tightly associated with increased cell number^19^, and this has also been observed in maize with changes in the auxin response^20^. In addition, increases in cell size have been found to have a proportional effect on leaf size, meaning that different parental inbred lines can make distinct contributions to heterosis^21^. Nevertheless, relatively large leaves contribute to hybrids having a higher photosynthesis capacity compared with that of their parents, with more total chlorophyll and higher photosynthesis carbon fixation capacity^19,21^.

Chinese cabbage (*Brassica rapa* L. subsp. *pekinensis*) is a major leafy crop species grown worldwide, F_1_ hybrids of which have been widely used in production for more than 30 years^22^. The developmental process is very similar between Chinese cabbage and Arabidopsis except for the leaf heading morphotype, which was domesticated approximately 500 years ago^23^. Several studies have been performed on *Brassica* species, including genetic distance, combining ability, and transcriptional and epigenetic analyses^12,24–28^. In Chinese cabbage and pak choi, the increase in photosynthesis is crucial to the formation of heterosis;^25–28^ furthermore, several genes have been identified, including the light-harvesting complex of photosystem II (*LHC*) and *CIRCADIAN CLOCK ASSOCIATED 1* (*CCA1*) genes^28^. However, little is known about its underlying molecular basis. Therefore, to understand the gene regulatory networks underlying leaf development and biomass heterosis in Chinese cabbage, it is important to thoroughly investigate the gene expression changes and posttranscriptional mechanisms that occur during specific developmental stages.

The current study aims to identify heterosis-regulating miRNAs and genes in Chinese cabbage and to verify the target transcripts by transcriptome-based degradome analysis. In this study, we used the F_1_ hybrid cultivar (F_1_ hereafter) Xin No. 3, which is one of the most popular varieties planted in China. Due to its high yield and good nutritional properties, Xin No. 3 accounts for 90 and 50% of the autumn planting area in Beijing and North China, respectively^29^. We found that the expression levels of the majority of miRNA clusters in the F_1_ hybrid was lower than the MPVs. Furthermore, 1819 target genes were identified via degradome sequencing. Transcriptome analysis revealed that photosynthesis and chlorophyll synthesis hybrid-MPVs of the DEGs were significantly different between the F_1_ hybrid and its parents, with increased photosynthesis capacity and chlorophyll content. Consequently, *BrGRF4.2-* and bra-miR396-overexpressing Arabidopsis plants showed significant changes in leaf size. Overall, we report miRNAs and genes that are thought to play a role in heterosis for Chinese cabbage biomass and provide new insight into this complex trait, thereby aiding crop improvement programs.

## Results

### Hybrids show significant heterosis compared to that of the parents during development

**Fig. 1:**
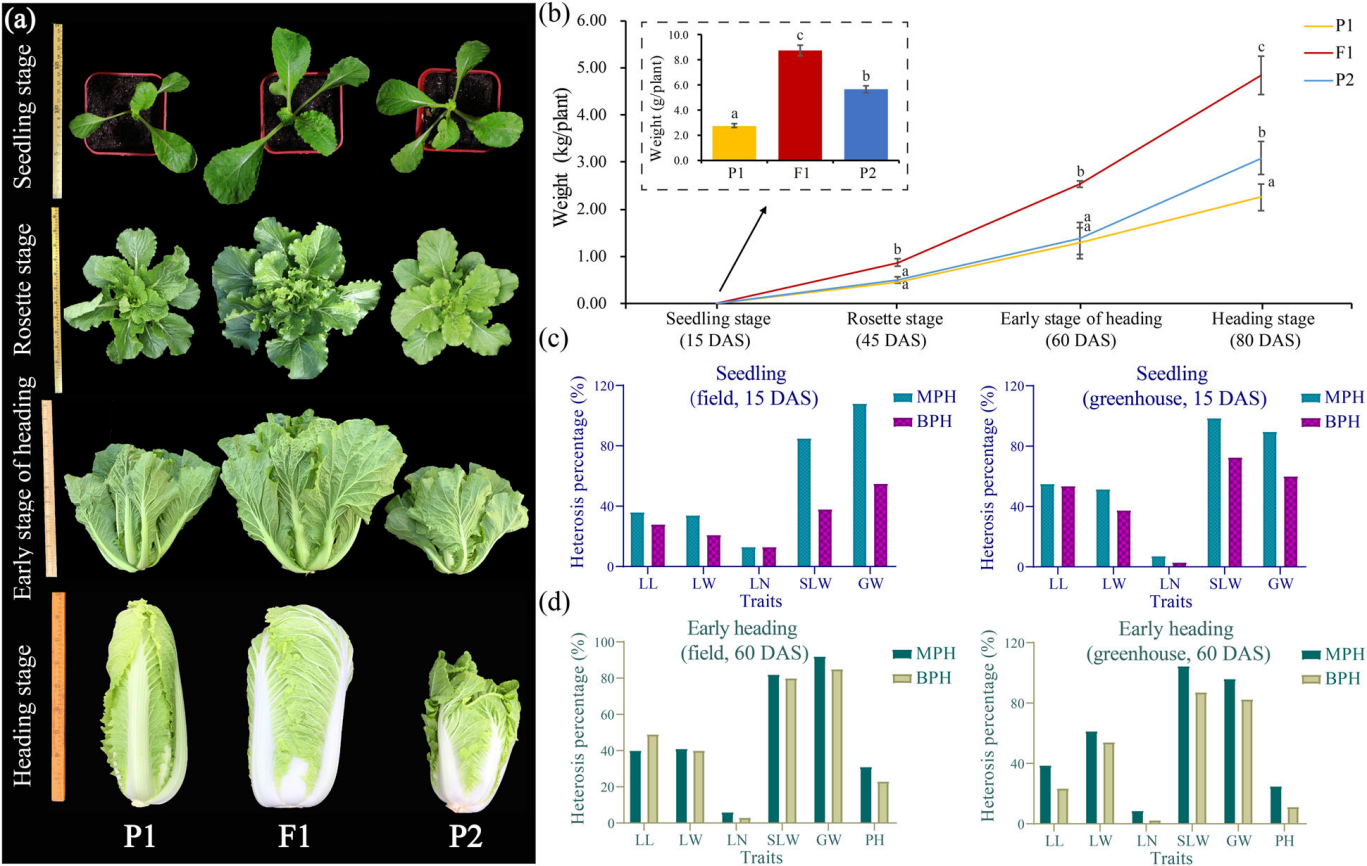
Comparisons of the growth and heterosis of a Chinese cabbage F_1_ hybrid and its inbred parent lines. **a** Phenotypes of the F_1_ hybrid and inbred parent lines at four vegetative growth stages. **b** Biomass (gross weight) of the F_1_ hybrid and its parental lines at four stages in the open field. The letters above the bars indicate significant differences at *P* < 0.05 (Student’s *t*-test) (Use the same statistical method hereafter). **c** The MPH and BPH of each trait for Xin No. 3 plants grown both in the open field (left) and in the greenhouse (right) at the S stage (15 days after sowing (DAS)). **d** The MPH and BPH of each trait for Xin No. 3 plants grown both in the open field (left) and in the greenhouse (right) at the H stage (60 DAS). GW, PH, SLW, LN, LL, and LW represent gross weight, plant height, single-leaf weight, leaf number, leaf length, and leaf width, respectively

The Chinese cabbage F_1_ hybrid Xin No. 3, derived from a cross of the inbred lines SD (P_1_) and JEY (P_2_), exhibits strong heterosis for biomass during vegetative growth and development (Fig. 1a). The significantly increased biomass of the F_1_ hybrid was first observed at the seedling stage (S), and it further increased at the early-heading stage (H) (Fig. 1b). We therefore used tissue from plants at two important vegetative stages (S and H) to determine the potential molecular basis underlying heterosis of Chinese cabbage.

Mid-parent heterosis (MPH) and better-parent heterosis (BPH) were used to evaluate biomass in terms of single-leaf weight, leaf number, leaf length, leaf width, plant height, and gross weight at the S and H stages in both the open field and greenhouse, respectively. The MPH and BPH in the open field were consistent with the values obtained in the greenhouse (Fig. 1c, d). Notably, we found strong positive MPH and BPH for gross weight (90–108% S-MPH, 55–60% S-BPH, 92–96% H-MPH, and 83–85% H-BPH) and single-leaf weight at both growth stages (Fig. 1c, d). In addition, positive MPH and BPH values for leaf length, leaf width, plant height, and leaf number were also observed for Chinese cabbage (Fig. 1c, d). Positive correlations were observed among gross weight, single-leaf weight, leaf width, and leaf length (correlation coefficients *r* = 0.46–0.94; Supplementary Fig. S1), while negative correlations were observed between leaf number and the other traits (Supplementary Fig. S1). These results indicate that the increased biomass yield observed for the Chinese cabbage F_1_ hybrid is mainly due to significantly increased leaf weight (a function of increased leaf length and leaf width) rather than leaf number.

### Increased numbers of nonadditively repressed miRNAs in the Chinese cabbage F_1_ hybrid

**Fig. 2:**
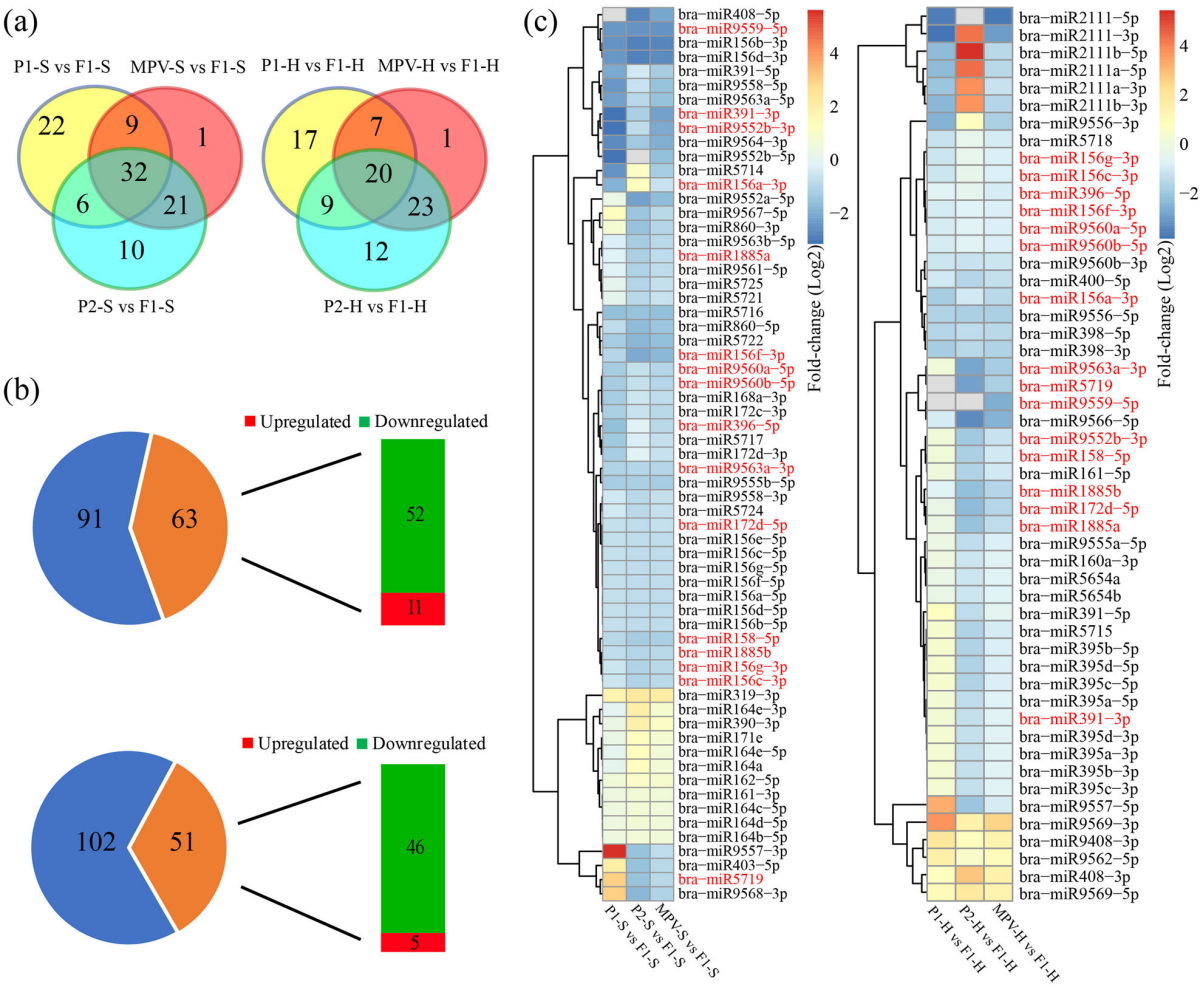
Overview of differentially expressed miRNAs (DEMs) in the F_1_ hybrid and the mid-parent value (MPV). **a** Venn diagram analyses of the total numbers of DEMs between F_1_, P_1_, and P_2_ at the S stage (left) and the H stage (right). **b** Statistics of up- or downregulated MVP-DEMs at the seedling stage (top) and early-heading stage (bottom). **c** Heatmaps showing relative fold-changes in MVP-DEM expression in the F_1_ hybrid compared with *P*_1_ values, *P*_2_ values, and MPVs at the S stage (left) and the H stage (right). The MVP-DEMs detected at both the S and H stages are marked in red

To characterize the potential roles of different miRNAs during heterosis in Chinese cabbage, we compared the dynamic changes in miRNA expression profiles between the two parents and the resulting hybrid at the S and H stages. A total of 154 known miRNAs among 30 miRNA families were identified in the hybrid and two parental lines. At a significance level of *P* ≤ 0.05 and a fold-change of ≥1.5, 130 DEMs were identified comparing the three genotypes at the S and H stages. When F_1_ values were compared with the *P*_1_ values, *P*_2_ values, and MPVs, 69, 69, and 63 DEMs were identified at the S stage, revealing 101 nonoverlapping DEMs (Fig. 2a). Additionally, 53, 64, and 51 DEMs were identified in the three comparisons at the H stage, revealing 89 DEMs without overlap (Fig. 2a). All DEMs in each comparison between the F_1_ values, MPVs, and two parental inbred line values are provided in Supplementary Table S1.

The miRNAs in the F_1_ hybrid showing significantly different expression levels compared to the MPVs (*P* ≤ 0.05 and fold-change ≥1.5) were designated hybrid MPV differentially expressed miRNAs (MVP-DEMs). Since these miRNAs were potentially responsible for generating the heterosis phenotype, MPV-DEMs were subsequently compared between the hybrid and two parental lines. Overall, 63 and 51 MPV-DEMs were identified at the S and H stages, respectively (Fig. 2b). Interestingly, of the 63 S-stage MPV-DEMs, 52 were nonadditively repressed in Xin No. 3, while 11 were nonadditively activated (Fig. 2b, c). Furthermore, we identified 46 nonadditively repressed MPV-DEMs and only five nonadditively activated MPV-DEMs at the H stage (Fig. 2b, c). This suggests that, compared with its parents, the F_1_ hybrid has more nonadditive miRNAs, most of which were nonadditively repressed. Moreover, 17 MPV-DEMs were identified at both the S and H stages (Fig. 2c).

### Low parental ELD of miRNAs in Chinese cabbage

**Fig. 3:**
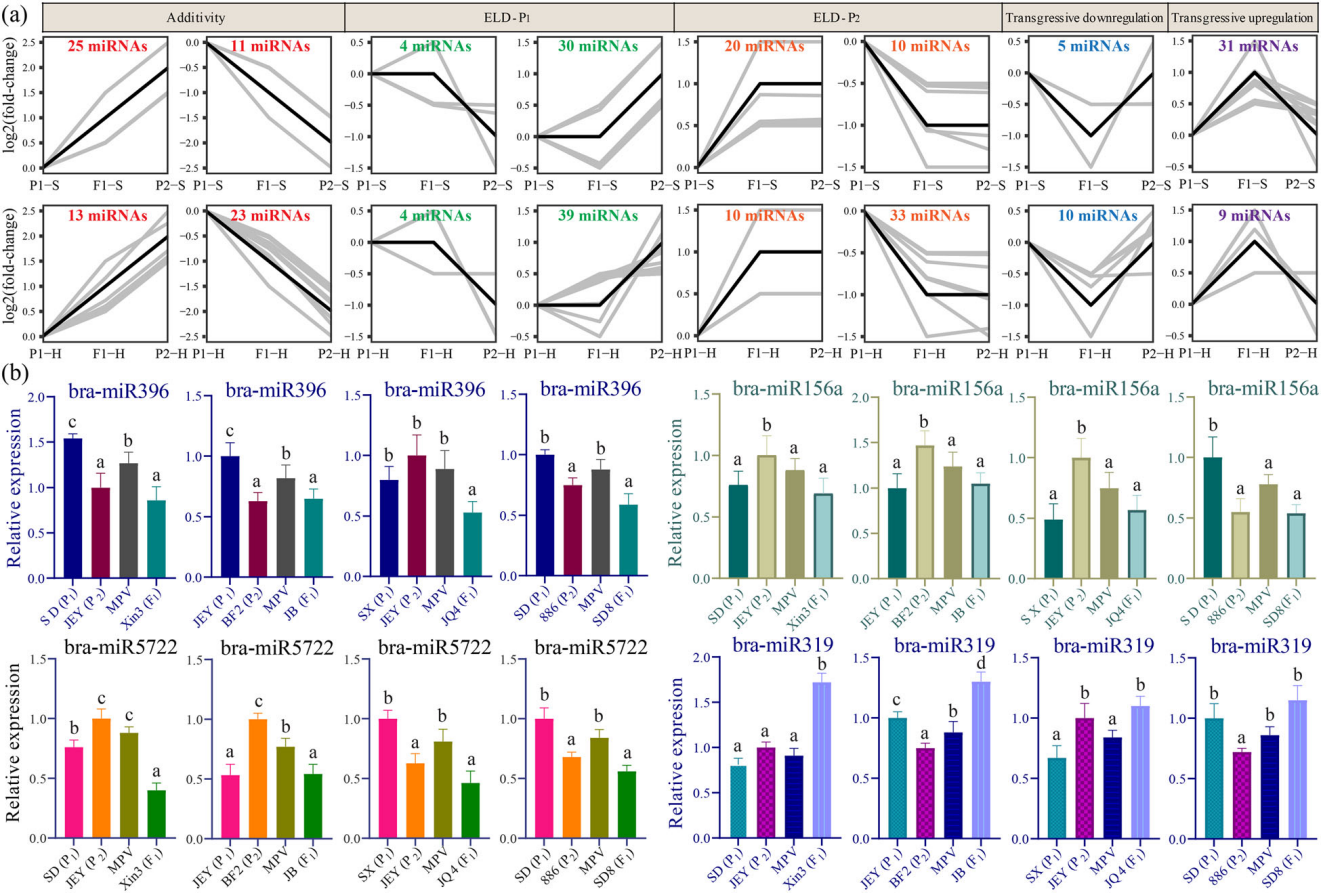
Expression patterns of differentially expressed miRNAs (DEMs) in a Chinese cabbage F_1_ hybrid. miRNAs with similar expression levels in the F_1_ hybrid and the P_1_ and P_2_ parents were designated ELD-P_1_ and ELD-P_2_, respectively. **a** Expression patterns of DEMs at the S stage (top row) and H stage (bottom row). The numbers of DEMs in each of the eight expression types are shown in the figure. **b** qRT-PCR validation of the expression levels of leaf development-related miRNAs (bra-miR396, bra-miR156a, bra-miR5722, and bra-miR319) in four Chinese cabbage F_1_ hybrids and their respective parental inbred lines, as well as the MPVs

To determine miRNA expression patterns, we classified genes into eight P_1_-hybrid-P_2_ (P_1_-H-P_2_) expression patterns for the S and H stages (Fig. 3a and Supplementary Table S2), as described by Shen et al. (2017). miRNAs whose expression levels in the F_1_ were statistically similar to those in the parents were subsequently designated parental ELD miRNAs. Large numbers of ELD miRNAs were revealed at the S (64) and H (86) developmental stages (Supplementary Table S2). Of these ELD miRNAs, most were expressed at levels similar to those of the parent associated with the low values, in which 40 (S) and 72 (H) low-ELD miRNAs were detected. Known miRNAs, such as bra-miR156, bra-miR319, bra-miR391, and bra-miR396, were all low-ELD miRNAs. In addition, three transgressive upregulated miRNAs (bra-miR168b, bra-miR168c, and bra-miR9555a) and one downregulated miRNA (bra-miR168a) were identified at both the S and H stages (Supplementary Table S2).

To verify the RNA sequencing results and confirm their general heterosis-related expression patterns in Chinese cabbage, we performed qRT-PCR analysis of four different hybrids and their five corresponding parents (two from the SD inbred line and three from the JEY inbred line) with four randomly selected miRNAs (bra-miR156a, bra-miR5722, bra-miR319, and bra-miR396). The results of the qRT-PCR assays showed that the differential expression of the miRNAs was in accordance with the sequencing results (Fig. 3b), indicating that the expression of low-ELD miRNAs might exhibit general dynamic changes in different Chinese cabbage hybrids compared with their parental lines.

### Degradome analysis of DEMs and their target mRNAs

**Fig. 4:**
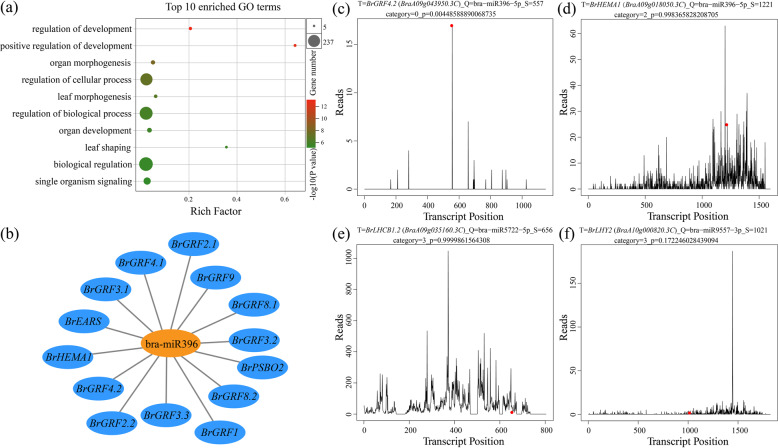
Target (T) plots of miRNAs verified by degradome sequencing and GO enrichment. **a** GO enrichment of the ELD-DEM target genes showing that ELD-DEMs function in leaf morphogenesis, organ development, and leaf shaping. **b** The bra-miR396 network and that of its targets. **c** T-plot showing that bra-miR396-5p cleaves the *BrGRF4.2* gene transcript. **d** T-plot showing that bra-miR396-5p cleaves the *BrHEMA1* gene transcript. **e** T-plot showing that bra-miR5722 cleaves the *BrLHCB1.2* gene transcript. **f** T-plot showing that bra-miR9557 cleaves the *BrLHY2* gene transcript. The red dots represent the expected cleavage positions on the target mRNAs

To reveal the target genes of the identified heterosis-related miRNAs in Chinese cabbage, we carried out degradome sequencing in this study. In total, 1819 target genes degraded by DEMs were identified (Supplementary Table S3). Based on functional enrichment analysis, the targets of the DEMs described above were significantly enriched in photosynthesis, nitrogen compound metabolic processes, chloroplast organization, and leaf development (Supplementary Fig. S2a). Moreover, 1488 target genes with 1665 target sites of the MPV-DEMs were identified (Supplementary Table S4) and significantly enriched in leaf morphogenesis, signal transduction, leaf shaping, and leaf development (Supplementary Fig. S2b). In addition, we identified 1219 target genes with 1343 target sites of the MPV-DEMs at the S stage and 841 target genes with 898 target sites of the MPV-DEMs at the H stage (Supplementary Table S5). In addition, the targets of low-ELD miRNAs were significantly enriched in genes involved in leaf morphogenesis and leaf shaping (Fig. 4a). These results suggest that DEMs, MPV-DEMs, and low-ELD miRNAs act in concert with each other to regulate the expression of genes involved in photosynthesis and leaf development during heterosis in Chinese cabbage.

In the Chinese cabbage degradome results, bra-miR396-5p, a miRNA known to be involved in leaf development, was notably verified to degrade 11 growth-regulating factors (GRFs) and many other functional genes (Fig. 4b and Supplementary Table S3). The degradome T-plot of *BrGRF3.1* and *BrGRF4.2*, which were identified as members of category 0^30^, showed a single clear peak at the degradation site (Fig. 4c and Supplementary Fig. S3). Importantly, the target gene of bra-miR5722 encodes chlorophyll a-b binding protein 1 (*BrLHCB1.2*) (Fig. 4d), and its homologous genes have previously been shown to play a role in regulating photosynthesis^28^. Additionally, bra-miR9557-3p was verified to degrade *LATE ELONGATED HYPOCOTYL* (*BrLHY2*), which interacts with EE- and CBS domain-containing downstream proteins involved in photosynthesis and starch metabolism (Fig. 4f). The T-plots for the remaining 12 targets of bra-miR396-5p are shown in Supplementary Fig. S3. We subsequently performed qRT-PCR analysis of *BrGRF4.2* and *BrLHCB1.2* in four different hybrids and their five corresponding parents. The results showed that the expression patterns of the target genes in different hybrid combinations were essentially consistent (Supplementary Fig. S4).

### Differentially expressed genes in the F_1_ hybrid and parental lines

**Fig. 5:**
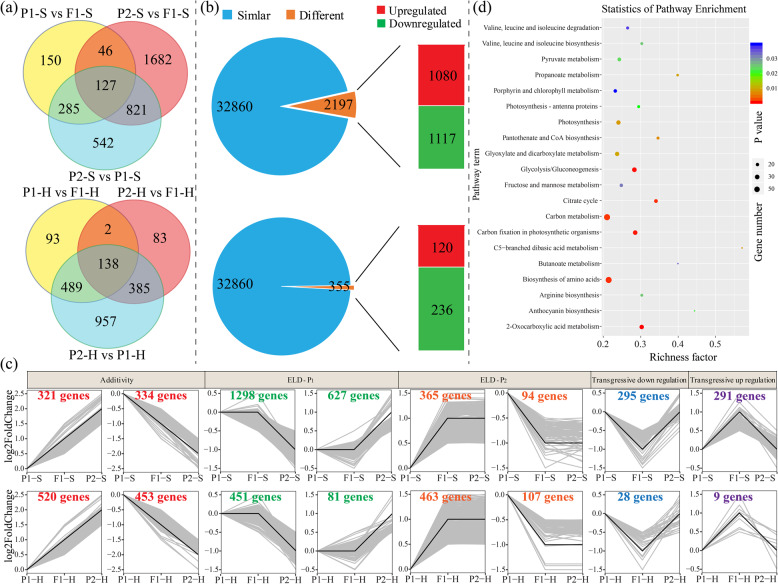
Overview of differentially expressed genes in an F_1_ hybrid and its parents at two developmental stages. **a** Venn diagram analyses showing the total numbers of DEGs in comparisons between the F_1_, P_1_, and P_2_ at the S stage (top) and H stage (bottom). **b** Genes whose expression was up- and downregulated at the S stage (top) and the H stage (bottom). **c** Expression patterns of eight groups of differentially expressed genes in the F_1_ hybrid and its parents at the S stage (top row) and H stage (bottom row). Genes showing a similar expression level in the F_1_ hybrid and the P_1_ and P_2_ parents were designated ELD-P_1_ and ELD-P_2_, respectively. **d** KEGG enrichment of parental expression level dominance genes

Dynamic changes in the transcriptomes of the F_1_ hybrid and its inbred parents were investigated by analyzing leaf transcripts at the S and H stages. A total of 3653 and 2146 DEGs were identified by comparing the three accessions at the S and H stages, respectively (Fig. 5a and Supplementary Table S6). At the S and H stages in the hybrid, there were 2197 (1080 up- and 1117 downregulated) and 356 (120 up- and 236 downregulated) genes (Fig. 5b and Supplementary Table S7) whose expression levels differed from the MPVs (MPV-DEGs), representing 1.1–6.7% of the expressed genes; therefore, these genes were considered to be related to heterosis. GO analysis revealed that all MPV-DEGs were enriched in various functional categories, including photosynthesis, cell division, cell proliferation, and response to auxin (Supplementary Fig. S5), suggesting a combined role of each biological pathway in heterosis for Chinese cabbage biomass. However, only 417 DEM target mRNAs were differentially expressed between the two stages, representing 8.53% of the total DEGs (Supplementary Fig. S6). We also found that only 158 and nine MPV-DEGs were targets of MPV-DEMs at the S and H stages, respectively, with 90 and four of them being negatively expressed (Supplementary Table S8).

We then classified the expression of the DEGs into eight patterns (Fig. 5c and Supplementary Table S9), as described above. A total of 2384 ELD genes and 1103 ELD genes were identified at the S and H developmental stages (Supplementary Table S9), respectively. Of the genes found to exhibit ELD, some were expressed at the same level as that in the parent with the highest level of expression (high-parental ELDs; Fig. 5c), while others were expressed at the level of the parent showing the lowest level of expression (low-parental ELDs; Fig. 5c). At the S and H developmental stages, more than 70% (1663/2384) and 83% (915/1,103) of the ELD genes were high-parental ELDs, respectively. Among these genes, only 66 and 21 high-parental ELD genes were negatively correlated with 26 and 15 low-ELD miRNAs, respectively (Supplementary Table S10). KEGG functional analysis of the high-parental ELD genes revealed significant enrichment in various functional categories, including porphyrin and chlorophyll metabolism, photosynthesis—antenna proteins, carbon metabolism, and carbon fixation in photosynthesis organisms (Fig. 5d).

### Photosynthesis capacity and chlorophyll content increased in the leaves of the Chinese cabbage F_1_ hybrid

**Fig. 6:**
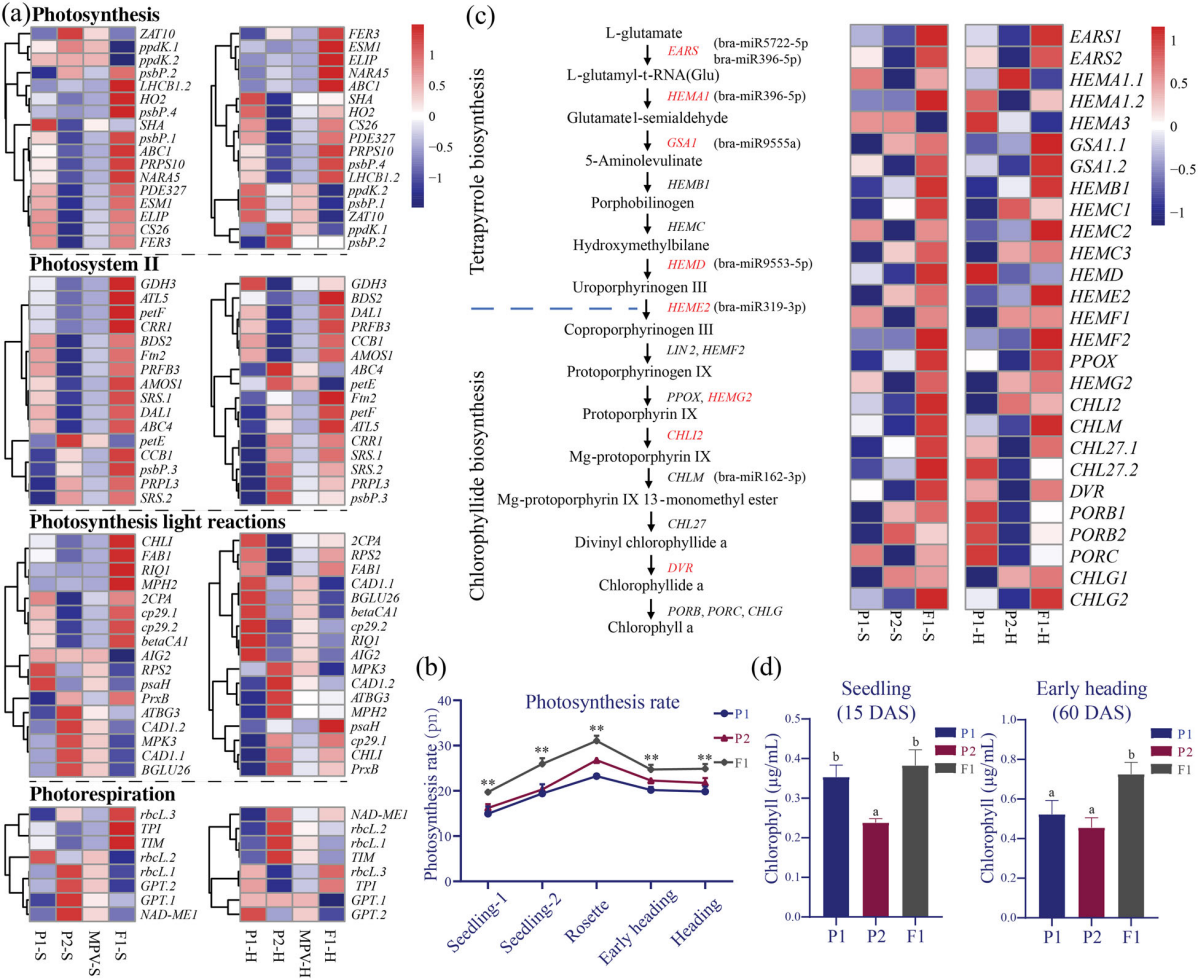
Differentially expressed genes involved in photosynthesis and chlorophyll biosynthesis. **a** Heatmaps showing relative changes in the expression of the DEGs involved in photosynthesis in F_1_, P_1_, and P_2_. **b** Photosynthesis rates of the F_1_ hybrid and its representative parental lines (P_1_ and P_2_) at five stages of Chinese cabbage development. Significant differences between the hybrids and P_2_ were calculated using Student’s *t*-test; **P < 0.01. **c** Heatmaps showing the fold-changes in the expression of genes involved in the tetrapyrrole and chlorophyllide biosynthesis pathways in the F_1_ hybrid, P_1_ and P_2_ at the S stage and H stage. Genes in the F_1_ hybrid whose expression was upregulated compared with that in the parental lines are shown in red. The expression levels were verified via qRT-PCR. **d** Chlorophyll contents of the Chinese cabbage F_1_ hybrid and the parental lines at the S stage (left) and H stage (right)

Transcriptome analysis revealed the photosynthesis genes that are enriched in the Chinese cabbage F_1_ hybrid. We further analyzed these genes and separated them based on their GO categories. In total, 52 S-MPV genes and eight H-MPV genes involved in photosynthesis-related biosynthesis were identified at the two developmental stages (Fig. 6). At the S stage, there were 37 genes whose expression was upregulated and 15 genes whose expression was downregulated, while three genes whose expression was upregulated and five genes whose expression was downregulated were identified at the H stage. The miRNAs that target these photosynthesis genes were subsequently identified (Supplementary Table S11). Importantly, the expression of *BrLHCB1.2*, the target of bra-miR5722, was upregulated at both the S and H stages in the hybrid, meaning that the improvement in photosynthesis capacity may contribute to heterosis in Chinese cabbage (Fig. 6a). Furthermore, the expression between the bra-miR5722 and *BrLHCB1.2* pairs was negatively correlated (correlation coefficient *r* = −0.62; Supplementary Fig. S7). To verify our findings, we compared the rate of photosynthesis per unit area between the F_1_ hybrid and its parents. As expected, the rate of photosynthesis per unit area was higher in the hybrid than in the parents from the seedling stage to the heading stage (Fig. 6b).

Increased chlorophyll content can result in increased photosynthesis^21^. In this study, we identified 27 Arabidopsis orthologous genes involved in tetrapyrrole and chlorophyll biosynthesis based on previous research^21^ and analyzed their transcript levels at both the S and H stages. We identified seven MPV genes and one MPV gene whose expression was upregulated and downregulated, respectively, at the S stage of the F_1_ hybrid (Fig. 6c), while no MPV genes were found at the H stage. However, the FPKM values showed that the expression of several genes was upregulated, even if the levels did not reach the MPV criterion (Fig. 6c). The miRNAs for these chlorophyll genes were subsequently identified (Fig. 6c). We also measured the chlorophyll content per gram of fresh weight in the F_1_ hybrid and the parents at the S and H stages (Fig. 6d) and found that the total chlorophyll content was greater in the hybrid than in the parents (Fig. 6d). These findings indicate that the higher chlorophyll content in the hybrid compared with its parents leads to increased biomass of the hybrid.

### Expression of genes controlling leaf size differs in the Chinese cabbage F_1_ hybrid

**Fig. 7:**
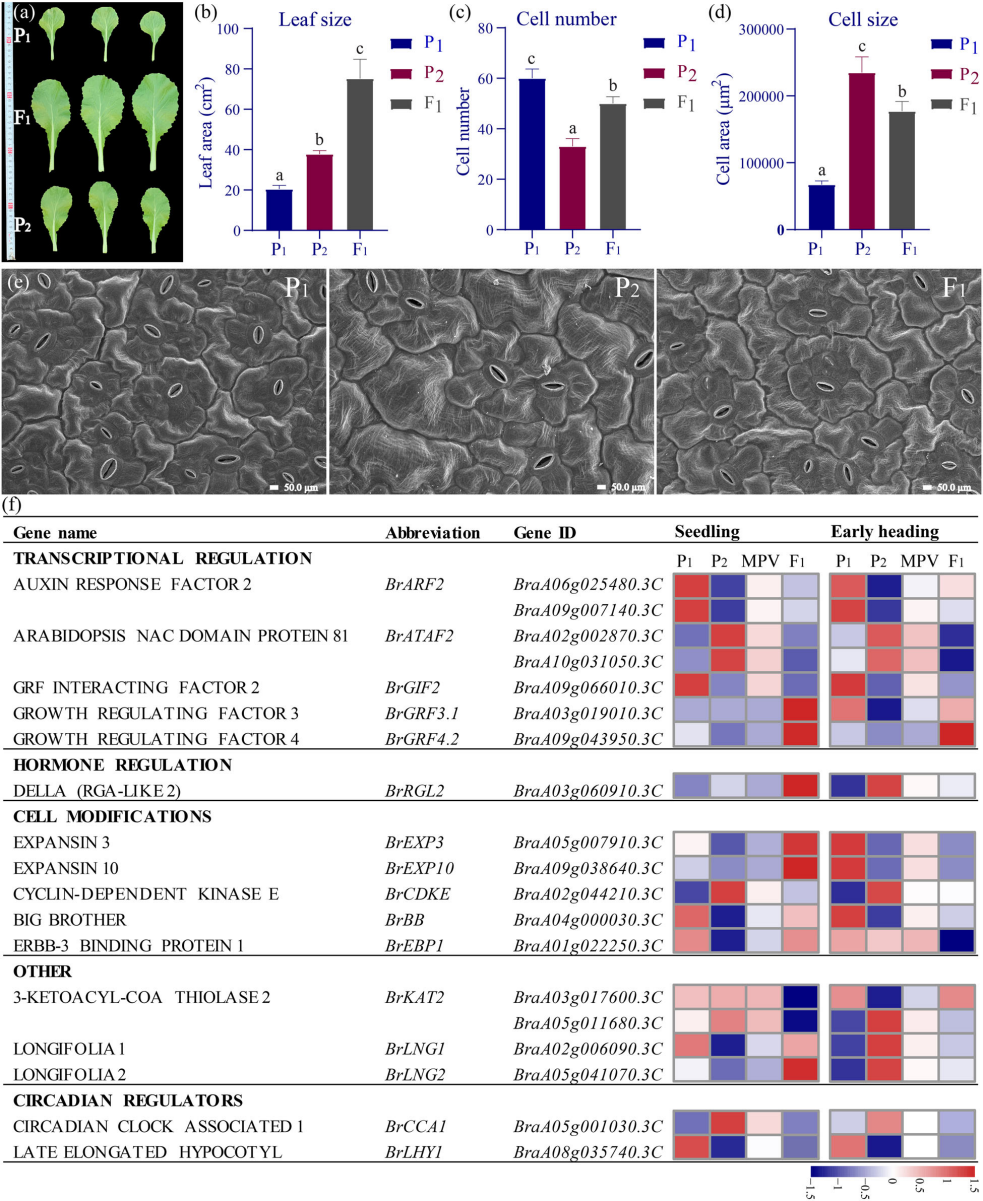
Comparisons of leaf size and expression levels of 19 genes involved in regulating leaf size. **a** Leaves of the Chinese cabbage F_1_ hybrid and its parental lines at 30 DAS. **b** Leaf area, **c** number of cells per leaflet (600x), and **d** cell size in the F_1_ hybrid and parental lines (P_1_ and P_2_). **e** Microscopy images of leaf cells from P_1_, P_2_ and F_1_ at 600x magnification. **f** Heatmaps showing the relative expression of 19 genes from five broad functional categories in P_1_, P_2_, and F_1_, as well as the MPVs

At the seedling stage (30 DAS), the leaf area was significantly larger for the Chinese cabbage F_1_ hybrid than for the parents due to increases in both leaf length and width (Fig. 7a, b), with strong positive MPH and BPH values (158% and 99.1%, respectively). The contribution of cell number and cell size in the larger leaves of the hybrid was therefore examined via electron microscopy (Fig. 7e). We counted the number and measured the size of epidermal cells at 600× magnification in the F_1_ hybrid and its parents and found that the cell number and size in the hybrid were intermediate between those of the two parents (Fig. 7c, b). These data indicate that the larger leaves of the hybrid are mainly caused by an increase in total cell number of the leaves.

Thus, we identified 112 homologs of 71 Arabidopsis genes^31,32^ involved in transcription, hormone regulation, and cell modification and then checked their expression levels in the F_1_ hybrid. Of these 112 genes, 19 were differentially expressed at the S or H stages (Fig. 7f). For most of these 19 genes, which are involved in several different regulatory networks, the changes in expression required to produce a larger leaf matched those observed in the F_1_ hybrid. The expression of *BrLHY1* and the circadian gene *BrCCA1*, the levels of which were below the MPV in all the hybrids, resulted in upregulated expression of downstream genes containing EE and CBS domains and involved in photosynthesis and carbohydrate metabolism, thereby increasing chlorophyll synthesis and starch metabolism^33^. The expression level of *BrARF2*, a repressor of cell division and leaf growth, was downregulated compared with the MPV at the seedling stages, while it showed an expression level similar to the MPV at the H stage (Fig. 7f). Furthermore, the expression level of *BrGRF4.2*, a positive regulator of both cell proliferation and cell enlargement, was upregulated in the hybrid compared with the MPV (Fig. 7f). However, the relative expression of several genes was opposite the phenotype or showed different patterns at different stages, indicating that increases in leaf size are regulated by multiple genetic pathways.

### Overexpression of bra-miR396 and *BrGRF4.2* affects leaf development

**Fig. 8:**
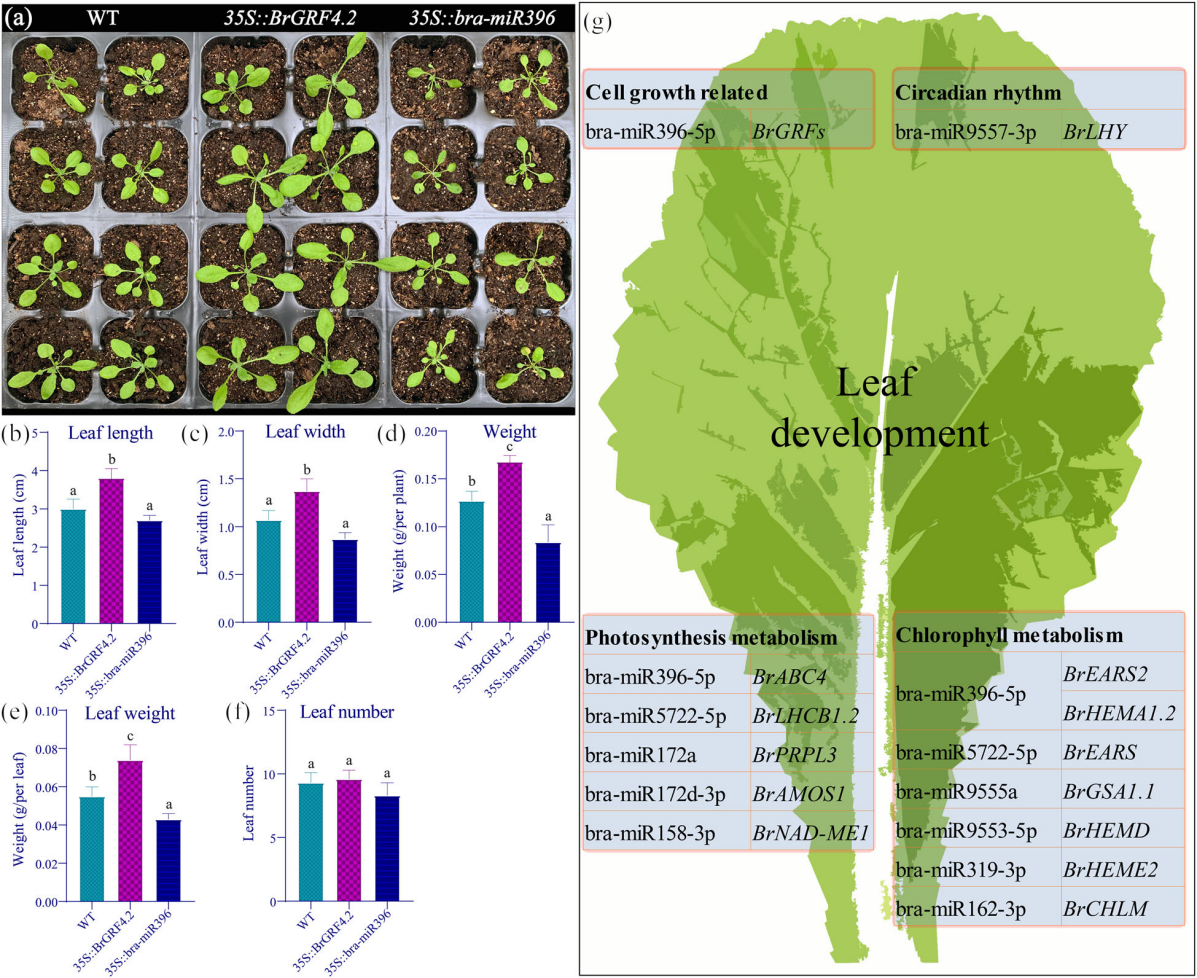
Phenotypes of transgenic Arabidopsis plants and effects of heterosis-related miRNAs in regulating leaf development. **a** Phenotypes of 25-day-old WT, *35::BrGRF4.2*, and *35S::bra-miR396* transgenic plants grown under 16 h light/8 h dark conditions. **b**–**f** Statistics of leaf length (**b**), leaf width (**c**), fresh weight (**d**), leaf weight (**e**), and leaf number (**f**). **g** Proposed model for the role of heterosis-related miRNAs in the regulation of leaf development in F_1_ hybrid Chinese cabbage

To further understand the function of the miR396 and *GRF* regulatory mechanisms in Chinese cabbage, we constructed bra-miR396 and *BrGRF4.2* overexpression vectors driven by an enhanced CaMV 35S promoter, after which the vectors were transformed into Arabidopsis. Compared with those of the wild-type (WT) control lines, the leaves of transgenic plants overexpressing *BrGRF4.2* were significantly enlarged, while bra-miR396-overexpressing plants had notably smaller leaves at 25 DAS (Fig. 8a). In general, the width and length of the *35::BrGRF4.2* plant leaves increased by approximately 28 and 27% (Fig. 8b, c), respectively, and the leaf weight and plant fresh weight were nearly 39 and 32% greater, respectively, than those of WT plants (Fig. 8d, e). In addition, the width and length of the leaves were reduced by approximately 20 and 10% (Fig. 8b, c), respectively, in *35S::bra-miR396* plants, and the leaf weight and the plant fresh weight were reduced by nearly 23 and 33%, respectively, compared with those of the WT plants (Fig. 8d, e). However, leaf numbers were not significantly different in the two transgenic lines compared to the control (Fig. 8f).

## Discussion

### Chinese cabbage as a model vegetable species for studies on biomass heterosis

Heterosis is a persistent mystery in biology and has been recognized for centuries^34^. Modern technologies, such as genomics and transcriptomics, are now used to identify heterosis-related genes that lead to increased crop yields, but the findings have been inconsistent. Since heterosis is a quantitative trait affected by multiple aspects, genomic analysis alone is not enough^9^. Researchers have focused on traits such as grain and fruit yield to interpret the possible molecular mechanisms for heterosis in crop species such as rice^35,36^, maize^37,38^, wheat^14,39^, rapeseed^12^, and tomato^40^. Indeed, biomass heterosis has been extensively studied in Arabidopsis^13,41^, whereas few studies of biomass heterosis have been conducted in vegetable crop species.

Chinese cabbage varieties have superior biomass yields compared with that of their progenitors. At present, the genetic basis of heterosis for Chinese cabbage biomass is still unclear, although F_1_ hybrids have been widely used in commercial production for more than 30 years^22^. To date, only a few studies have focused on the potential mechanisms underlying heterosis for Chinese cabbage biomass, and the role of different traits in biomass heterosis is still unclear. In this study, we confirmed the significant contributions of leaf weight, leaf width, and leaf length to biomass heterosis. The increased biomass of the F_1_ was not the result of increased leaf number, because the leaf number of the F_1_ plants was found to be similar to that of the two parents. However, leaf weight is a complex quantitative trait influenced by leaf blade weight, petiole weight, leaf size, and leaf thickness^42^, and more-detailed studies are needed.

### miRNAs are important regulators of heterosis for Chinese cabbage biomass

miRNAs involved in plant growth and vigor are known to be differentially regulated in hybrids relative to their parents, affecting the expression patterns of respective targets during metabolism^1^. In Arabidopsis, wheat, and *B. napus* hybrids, a large number of well-known miRNAs are nonadditively expressed, leading to nonadditive expression of their targets, which can affect growth^12–14^. The observation that some miRNAs also exhibit nonadditive expression during the seedling and early-heading stages indicated that the heterosis expression patterns of miRNAs are similar in different species. Furthermore, the expression of a large proportion of nonadditively expressed miRNAs was found to be repressed in Chinese cabbage F_1_ hybrids, while only 79 and four upregulated targets were identified (Supplementary Table S8). This is consistent with what has been reported in strawberry and *B. napus* studies^12,43^, demonstrating that the expression of target mRNAs may also be modulated by other regulators or methylation-type modifications.

Leaf development involves the complex regulation of different miRNA pathways that cooperate with each other to form a multilayer network. Our degradome data also show that the targets of the DEMs in this study are enriched in leaf morphogenesis and leaf shaping. During leaf development in Arabidopsis, miR396 expression increases, while that of the target gene *GRF* decreases, acting as an important indicator of leaf size^44^. Previous studies have shown that miR156, miR164, and miR319 also act as key regulators of leaf development^15,17^ by targeting multiple *SPL*, *CUC*, and *TCP* genes, respectively. However, we did not detect the expected *SPL*, *CUC*, and *TCP* genes in our degradome data. It is therefore possible that the miR396-*GRF* pathway is significantly associated with leaf size heterosis in *B. rapa* hybrids.

Leaf morphogenesis is modulated by *GRF* genes, which play a role in cell division and expansion. Overexpression of *GRF1*/*2*/*3* resulted in larger leaves, while loss-of-function mutants had smaller leaves^44^. It has been shown that *GRF* interacting factor 1 (*GIF1*) interacts with GRF proteins to maintain cell proliferation in vegetative and primordial reproductive organs^45^. The *B. rapa* homologs of *AtGRF4* and *AtGIF2* were found to be nonadditively expressed in the F_1_, suggesting that the miR396-*GRF* pathway is conserved and potentially involved in heterosis for biomass during leaf development. Consequently, our bra-miR396 and *BrGRF4.2* overexpression results also revealed functions similar to those of the Arabidopsis genes during leaf development.

### Multiple metabolic pathways are associated with heterosis of Chinese cabbage

The larger leaves of the F_1_ hybrids lead to increased photosynthesis and energy production, both of which have important effects on hybrid growth and yield^21,26–28^. This is obvious in hybrids of Arabidopsis, Chinese cabbage, and pak choi, where the greater capacity for photosynthesis increases under high light^21,26–28^. Furthermore, previous reports have shown that the DEGs were enriched in the photosynthesis, thylakoid, chloroplast, and hormone categories^26,28^. This coincides with our observations in *B. rapa* that the photosynthesis rate and chlorophyll content of the F_1_ hybrid increased due to transcriptional and posttranscriptional factors. Increased expression of the chlorophyll gene *LHCB* is associated with larger cells and an increased number of granum thylakoids^28^, while damage to chloroplasts treated with norflurazon leads to a decrease in cell size^46^. One *B. rapa* homolog of *AtLHCB* was found to be nonadditively upregulated in the F_1_, and this gene is the target of the nonadditively repressed bra-miR5722 (Fig. 8g and Supplementary Fig. S7), which may provide new insight into the regulation of *BrLHCB*.

Changes in the circadian rhythm have been shown to provide the energy production needed to support increased hybrid growth^19,33^. The circadian rhythm regulates many key processes, including photosynthesis and nighttime starch use, and its oscillation patterns are different between parents, with changes in these patterns affecting the formation of heterosis^33^. At both the S and H stages of the hybrid, we found that key circadian clock regulatory genes show patterns and levels of expression that differ from those of the parents in the daytime. Although we did not have nighttime transcription data, daytime changes in the expression of circadian clock genes in Arabidopsis F_1_ hybrids and allopolyploids were similar to those in the Chinese cabbage hybrid. The circadian clock is under miRNA control^12,33^, suggesting that these circadian regulators may be caused by posttranscriptional changes in hybrids.

## Conclusion

In conclusion, our study revealed the importance of combining miRNA data and transcriptome analysis data to determine the comprehensive factors related to heterosis for biomass. We identified several miRNAs in the F_1_ hybrids that play important roles in regulating target genes associated with photosynthesis, leaf development, and plant growth. Taken together, the results provide new insight into the key regulatory networks and genes related to leaf development during heterosis of Chinese cabbage.

## Materials and Methods

### Plant materials and phenotyping

For the heterosis study, we used two inbred parental lines, SD (P_1_), JEY (P_2_), and their F_1_ hybrid (Xin No. 3). The first leaf (counted from the edge) at the seedling stage (15 DAS) and the fifth leaf (from the edge) at the early-heading stage (60 DAS) were used in all experiments. Total DNA and RNA samples were extracted from three replicates.

The phenotypes were evaluated during the day at two locations (field and greenhouse) (three replicates each) in 2018. Whole-plant gross weight (GW), single-leaf weight (SLW), leaf number (LN), leaf length (LL), leaf width (LW), and plant height (PH) were measured at 15 and 60 DAS, respectively. The fifth outside leaf was chosen for measurements in our study. Five plants were measured for each genotype. The BPH was calculated as BPH = (F_1_ – BP)/BP, where F_1_ is the hybrid and BP is the better-performing parental line. MPH was calculated using the equation MPH = (F_1_ – MP)/MP, where F_1_ is the hybrid and MP is the mean of the two parental lines.

### Small RNA (sRNA) library construction and sequencing

sRNA libraries were constructed from the Chinese cabbage F_1_ hybrid Xin No. 3 and its parental lines SD and JEY. A total of 18 leaf samples (3 materials x 2 growth stages x 3 biological replicates) were prepared for sRNA analysis using a TruSeq Small RNA Sample Prep Kit (Illumina, San Diego, USA), as directed by the manufacturer. The RNA concentration was measured using a NanoDrop ND-2000 spectrophotometer (Thermo Scientific, Wilmington, DE, USA). Single-end sequencing (50 bp) was then conducted on a BGISEQ-500 platform (BGI, Wuhan, China).

### Identification of Chinese cabbage miRNAs via deep RNA sequencing

The raw deep-sequencing data were preprocessed to eliminate low-quality tags, yielding sRNA tags. The clean sRNA reads were then aligned to the GenBank and Rfam 12.2 (http://rfam.xfam.org/) databases using BLAST searches and bowtie^47^ to screen and remove sequences associated with other types of small RNAs (rRNA, scRNA, snoRNA, snRNA, and tRNA). The remaining sRNAs mapped to the *B. rapa* reference genome (V3.0) were used as search queries against the miRBase 21.0 database (http://www.mirbase.org/) to identify known miRNAs. Novel miRNAs were predicted using MIREAP software (http://sourceforge.net/projects/mireap/). Levels of miRNA expression were calculated using the transcripts per kilobase million (TPM) method. The R package DESeq2^48^ was used to identify DEMs by the use of negative binomial generalized linear models. For each conserved miRNA, MPVs were calculated based on comparable expression levels. miRNAs whose expression level in the F_1_ hybrid differed from the MPVs were then selected as candidate heterosis-related miRNAs.

### Degradome library construction, sequencing, and data processing

In accordance with the research of German et al.^49^, a degradome library was constructed using leaf samples (15 DAS) from the F_1_ hybrid Xin No. 3. Briefly, poly(A)-enriched RNA was captured by magnetic beads. Reverse transcription was then performed to generate first-strand cDNA using biotin-labeled random primers. The target sequences were then captured using magnetic beads instead of PAGE-gel purification. Finally, the purified cDNA library was sequenced on an Illumina HiSeq 2000 instrument (LC Sciences, Hangzhou, China). The categories of cleaved miRNA targets were identified and classified using the CleaveLand 3.0 pipeline^30^ and the ACGT301-DGE v1.0 program (LC Sciences, Hangzhou, China), after which T-plot figures were constructed.

### RNA sequencing

Total RNA was extracted from each sample using TRIzol reagent (Invitrogen, Life Technologies, USA) according to the manufacturer’s instructions. RNA-seq libraries were prepared using an Illumina standard mRNA-seq Library Preparation Kit and sequenced on an Illumina HiSeq 2000 instrument in paired-end mode. The NGS QC Toolkit v2.3.3^50^ was first used to discard paired-end reads containing adapters, reads with poly-Ns, or reads with more than 20% low-quality bases (PHRED-like score <20). All the clean reads were then mapped to the *B. rapa* genome sequence using HISAT v2.0.4^51^ with the default settings. After genome mapping, StringTie^52^ was used to reconstruct novel transcripts, which were then identified using the genome annotation information together with Cuffcompare^53^. DESeq2 version: 1.22.2^48^ was used to identify DEGs that had a fold-change of ≥1.5 and an adjusted *P* value of ≤0.05. GO functional enrichment was carried out using GOseq software^54^.

### Validation of miRNA and target gene expression via qRT-PCR analysis

Total RNA was extracted from the first outside leaf of four F_1_ hybrids and their five corresponding parents at the seedling stage (15 DAS) using a mirVana RNA Isolation Kit (Ambion, USA) as directed by the manufacturer.

Quantification was carried out according to a two-step reaction process: reverse transcription (RT) and PCR. The RT reaction and real-time PCR assay were conducted in accordance with published methods^55^. Three technical replicates for each biological reaction and three biological replicates were evaluated for each sample. The expression levels of the miRNAs were normalized to those of 5S rRNA and were calculated using the 2^−ΔΔCt^ method^56^. The sequences of all the miRNAs and gene-specific primers are listed in Supplementary Table S12 and Supplementary Table S13, respectively.

### Vector construction and plant transformation

To clone *BrGRF4.2* from Chinese cabbage, specific primers for *BrGRF4.2* were designed based on sequence information of the *B. rapa* reference genome^57^. The full-length cDNA of *BrGRF4.2* was amplified from Chinese cabbage total RNA using gene-specific PCR primers. The resulting PCR products were then cloned into a pMD18-T vector (Takara, Japan) for DNA sequencing. To construct *35S::BrGRF4.2* overexpression vectors, we amplified the entire 1149 bp coding sequence of *BrGRF4.2* and subcloned it into pCR8/GW/TOPO entry vectors (Invitrogen) according to the manufacturer’s instructions. The CDS fragment was then inserted into a pCAMBIA2300-EGFP vector through LR recombination reactions.

The 840 bp genomic fragment containing the precursor sequence of bra-miR396 (162 bp) was amplified from Chinese cabbage and then cloned into a pCAMBIA2300-EGFP vector under the control of the CaMV 35S promoter for bra-miR396 overexpression. The *35S::BrGRF4.2* and *35S::bra-miR396* plasmids were then introduced into *Agrobacterium tumefaciens* strain GV3101, which were subsequently transformed into Arabidopsis Col-0 plants according to the floral dip method^58^.

### Chlorophyll extraction and quantification

The first outside leaf at the seedling stage (15 DAS) and the fifth outside leaf at the early-heading stage (60 DAS) were ground in 80% (v/v) acetone, after which the absorbance of the supernatants was determined at 646.6 and 663.6 nm. The concentrations of chlorophyll a and b were then calculated as follows: chlorophyll a (μg/mL) = 12.25 × A_663.6_ − 2.55 × A_646.6_; chlorophyll b (μg/mL) = 20.31 × A_646.6_ − 4.91 × A_663.6_. The total chlorophyll content was then determined using the sum of chlorophyll a and chlorophyll b.

### Leaf size, cell size, cell number, and photosynthesis rate measurements

The first leaf of Chinese cabbage at 30 DAS was cut into thin (5 mm wide) slices and then processed in accordance with a previously reported method^59^. The tissues were then observed under a scanning electron microscope (Hitachi S-3400N). A similar region of each leaf was used for observations of epidermal cells of all the samples. Images were captured at 600x magnification, and the cell number per unit area was counted using ImageJ software to determine the average cell size. The number of epidermal cells for each leaf was also calculated.

The photosynthesis rate (Pn) of the first outside Chinese cabbage leaf was measured with a portable photosynthesis system (LI-6400; LI-COR, Inc., Lincoln, NE) at 15 DAS, 20 DAS, 45 DAS, 60 DAS, and 80 DAS. The light intensity was maintained at 100, 400, and 1200 μmol/m^2^/s, the CO_2_ was maintained at 380 to 400 μmol/mol, and the temperature was 25 °C.

## Supplementary information

Supplementary Figure S7. Correlation between bra-miR5722 and BrLHCB1.2

Supplementary Figure S1. Correlation between all traits in the F1 hybrid and its parental inbred lines

Supplementary Figure S2. GO enrichment analysis of the DEM (a) and MVP-DEM (b) target genes

Supplementary Figure S3. Target (T) plots of miRNAs validated by degradome sequencing

Supplementary Figure S4. The expression of miRNAs’ targets in four Chinese cabbage F1 hybrids, their respective parental inbred lines, and the MPV

Supplementary Figure S5. Functional annotation of enriched GO terms in the three main categories ‘biological process’, ‘cellular component’, and ‘molecular function’ for MPV-DEGs in Chinese cabbage

Supplementary Figure S6. Venn diagram representing the number of DEM’s targets and DEGs

Supplementary Table S1

Supplementary Table S2

Supplementary Table S3

Supplementary Table S4

Supplementary Table S5

Supplementary Table S6

Supplementary Table S7

Supplementary Table S8

Supplementary Table S9

Supplementary Table S10

Supplementary Table S11

Supplementary Table S12

Supplementary Table S13

## Data Availability

The sequencing data have been deposited in the NCBI Sequence Read Archive (SRA) under accession number PRJNA634805.

## Supplementary information

**Supplementary information** The online version contains supplementary material available at 10.1038/s41438-021-00474-6.

## References

[1] Chen ZJ (2013). Genomic and epigenetic insights into the molecular bases of heterosis. Nat. Rev. Genet..

[2] Schnable P, Springer N (2013). Progress toward understanding heterosis in crop plants. Annu. Rev. Plant Biol..

[3] Birchler J, Yao H, Chudalayandi S, Vaiman D (2010). & Veitia, R. Heterosis. Plant Cell.

[4] Bruce AB (1910). The mendelian theory of heredity and the augmentation of vigor. Science.

[5] East EM (1936). Heterosis. Genetics.

[6] Schnell F, Cockerham C (1992). Multiplicative vs. arbitrary gene action in heterosis. Genetics.

[7] Hochholdinger F, Höcker N (2007). Towards the molecular basis of heterosis. Trends Plant Sci..

[8] He G, Elling A, Deng X (2010). The epigenome and plant development. Annu. Rev. Plant Biol..

[9] Lippman Z, Zamir D (2007). Heterosis: revisiting the magic. Trends Genet..

[10] Jones-Rhoades MW, Bartel DP, Bartel B (2006). MicroRNAS and their regulatory roles in plants. Annu. Rev. Plant Biol..

[11] Voinnet O (2009). Origin, biogenesis, and activity of plant microRNAs. Cell.

[12] Shen Y (2017). Analysis of transcriptional and epigenetic changes in hybrid vigor of allopolyploid Brassica napus uncovers key roles for small RNAs. Plant J..

[13] Ha M (2009). Small RNAs serve as a genetic buffer against genomic shock in Arabidopsis interspecific hybrids and allopolyploids. Proc. Natl Acad. Sci. USA.

[14] Li A (2014). mRNA and small RNA transcriptomes reveal insights into dynamic homoeolog regulation of allopolyploid heterosis in Nascent Hexaploid Wheat. Plant Cell.

[15] Xu M (2016). Developmental functions of miR156-regulated SQUAMOSA PROMOTER BINDING PROTEIN-LIKE (SPL) genes in Arabidopsis thaliana. PLoS Genet..

[16] Ben-Gera H (2016). Auxin-mediated lamina growth in tomato leaves is restricted by two parallel mechanisms. Plant J..

[17] Bresso EG, Chorostecki U, Rodriguez RE, Palatnik JF, Schommer C (2018). Spatial control of gene expression by miR319-regulated TCP transcription factors in leaf development. Plant Physiol..

[18] Omidbakhshfard MA, Proost S, Fujikura U, Mueller-Roeber B (2015). Growth-regulating factors (GRFs): a small transcription factor family with important functions in plant biology. Mol. Plant.

[19] Groszmann M (2014). Intraspecific Arabidopsis hybrids show different patterns of heterosis despite the close relatedness of the parental genomes. Plant Physiol..

[20] Guo M (2013). Maize ARGOS1 (ZAR1) transgenic alleles increase hybrid maize yield. J. Exp. Bot..

[21] Fujimoto R, Taylor JM, Shirasawa S, Peacock WJ, Dennis ES (2012). Heterosis of Arabidopsis hybrids between C24 and Col is associated with increased photosynthesis capacity. Proc. Natl Acad. Sci. USA.

[22] Zhang SF, Song ZH, Zhao XY (1990). Breeding of interactive genic sterile line in chinese cabbage (Brassica pekinensis Rupr) and utilization model. Acta. Hort. Sin..

[23] Cheng F (2016). Subgenome parallel selection is associated with morphotype diversification and convergent crop domestication in Brassica rapa and Brassica oleracea. Nat. Genet..

[24] Li H (2018). Transcriptome and DNA methylome reveal insights into yield heterosis in the curds of broccoli (Brassica oleracea L var. italic). BMC Plant Biol..

[25] Saeki N (2016). Molecular and cellular characteristics of hybrid vigour in a commercial hybrid of Chinese cabbage.. BMC Plant Biol..

[26] Kong X (2020). Transcriptome analysis of biological pathways associated with heterosis in Chinese cabbage. Genomics.

[27] Yi H (2017). Genome-wide analysis of heterosis-related genes in non-heading Chinese cabbage. J. Plant Biotechnol..

[28] Liu T (2020). Enhanced photosynthetic activity in pak choi hybrids is associated with increased grana thylakoids in chloroplasts. Plant J..

[29] Su T (2018). Development of cost-effective single nucleotide polymorphism marker assays for genetic diversity analysis in Brassica rapa. Mol. Breed..

[30] Addo-Quaye C, Miller W, Axtell MJ (2008). CleaveLand: a pipeline for using degradome data to find cleaved small RNA targets. Bioinformatics.

[31] Gonzalez N, Beemster G (2009). David and Goliath: what can the tiny weed Arabidopsis teach us to improve biomass production in crops?. Curr. Opin. Plant Biol..

[32] Breuninger H, Lenhard M (2010). Control of tissue and organ growth in plants. Curr. Top. Dev. Biol..

[33] Ni Z (2009). Altered circadian rhythms regulate growth vigour in hybrids and allopolyploids. Nature.

[34] Darwin, C. *The Effects of Cross and Self-Fertilisation in the Vegetable Kingdom* (John Murray, 1876).

[35] Wang C (2019). Dissecting a heterotic gene through GradedPool-Seq mapping informs a rice-improvement strategy. Nat. Commun..

[36] Huang X (2016). Genomic architecture of heterosis for yield traits in rice. Nature.

[37] Liu H (2019). Genome‐wide identification and analysis of heterotic loci in three maize hybrids. Plant Biotechnol. J..

[38] Riedelsheimer C (2012). Genomic and metabolic prediction of complex heterotic traits in hybrid maize. Nat. Genet..

[39] Kenan-Eichler M (2011). Wheat hybridization and polyploidization results in deregulation of small RNAs. Genetics.

[40] Krieger U (2010). The flowering gene single flower truss drives heterosis for yield in tomato. Nat. Genet..

[41] Ng D (2014). A role for CHH methylation in the parent-of-origin effect on altered circadian rhythms and biomass heterosis in Arabidopsis intraspecific hybrids. Plant Cell.

[42] Han J, Hou X, Shi G, Geng J, Deng X (2007). Genetic model analysis of leaf-weight traits in non-heading Chinese cabbage (Brassica campestris ssp. chinensis Makino). Hereditas.

[43] Li D (2019). Integrated analysis of high-throughput sequencing data shows abscisic acid-responsive genes and miRNAs in strawberry receptacle fruit ripening. Hortic. Res..

[44] Rodriguez RE, Debernardi JM, Palatnik JF (2014). Morphogenesis of simple leaves: regulation of leaf size and shape. Wires. Dev. Biol..

[45] Debernardi J (2014). Post-transcriptional control of GRF transcription factors by microRNA miR396 and GIF co-activator affects leaf size and longevity. Plant J..

[46] Fhdiana I, Meehan L, Harkins K, Chory J, Rodermel S (1996). Lhcb transcription is coordinated with cell size and chlorophyll accumulation. Plant Physiol..

[47] Langmead B, Salzberg SL (2012). Fast gapped-read alignment with Bowtie 2. Nat. Methods.

[48] Love MI, Wolfgang H, Simon A (2014). Moderated estimation of fold change and dispersion for RNA-seq data with DESeq2. Genome Biol..

[49] German MA, Luo S, Schroth G, Meyers BC, Green PJ (2009). Construction of parallel analysis of RNA ends (PARE) libraries for the study of cleaved miRNA targets and the RNA degradome. Nat. Protoc..

[50] Patel RK, Jain M (2012). NGS QC Toolkit: a toolkit for quality control of next generation sequencing data. PLoS ONE.

[51] Kim D, Langmead B, Salzberg SL (2015). HISAT: a fast spliced aligner with low memory requirements. Nat. Methods.

[52] Mihaela P (2015). StringTie enables improved reconstruction of a transcriptome from RNA-seq reads. Nat. Biotechnol..

[53] Trapnell C (2010). Transcript assembly and quantification by RNA-Seq reveals unannotated transcripts and isoform switching during cell differentiation. Nat. Biotechnol..

[54] Young MD, Wakefield MJ, Smyth GK, Oshlack A (2010). Gene ontology analysis for RNA-seq: accounting for selection bias. Genome Biol..

[55] Li P (2020). Genome-wide analysis of mRNA and lncRNA expression and mitochondrial genome sequencing provide insights into the mechanisms underlying a novel cytoplasmic male sterility system, BVRC-CMS96, in Brassica rapa. Theor. Appl. Genet..

[56] Livak KJ, Schmittgen TD (2001). Analysis of relative gene expression data using real-time quantitative PCR and the 2(-Delta Delta C(T)) method. Methods.

[57] Wang X (2011). The genome of the mesopolyploid crop species Brassica rapa. Nat. Genet..

[58] Clough S, Bent A (1998). Floral dip: a simplified method for Agrobacterium-mediated transformation of Arabidopsis thaliana. Plant J..

[59] Li P (2018). BrLAS, a GRAS transcription factor from Brassica rapa, is involved in drought stress tolerance in transgenic Arabidopsis. Front. Plant Sci..

