# Supplementary figures and images for: Genome-wide analysis of changes in miRNA and target gene expression reveals key roles in heterosis for Chinese cabbage biomass
Source: Hortic Res. 2021 Mar 1;8:39. doi: 10.1038/s41438-021-00474-6 (PMC7917107; doi:10.1038/s41438-021-00474-6)

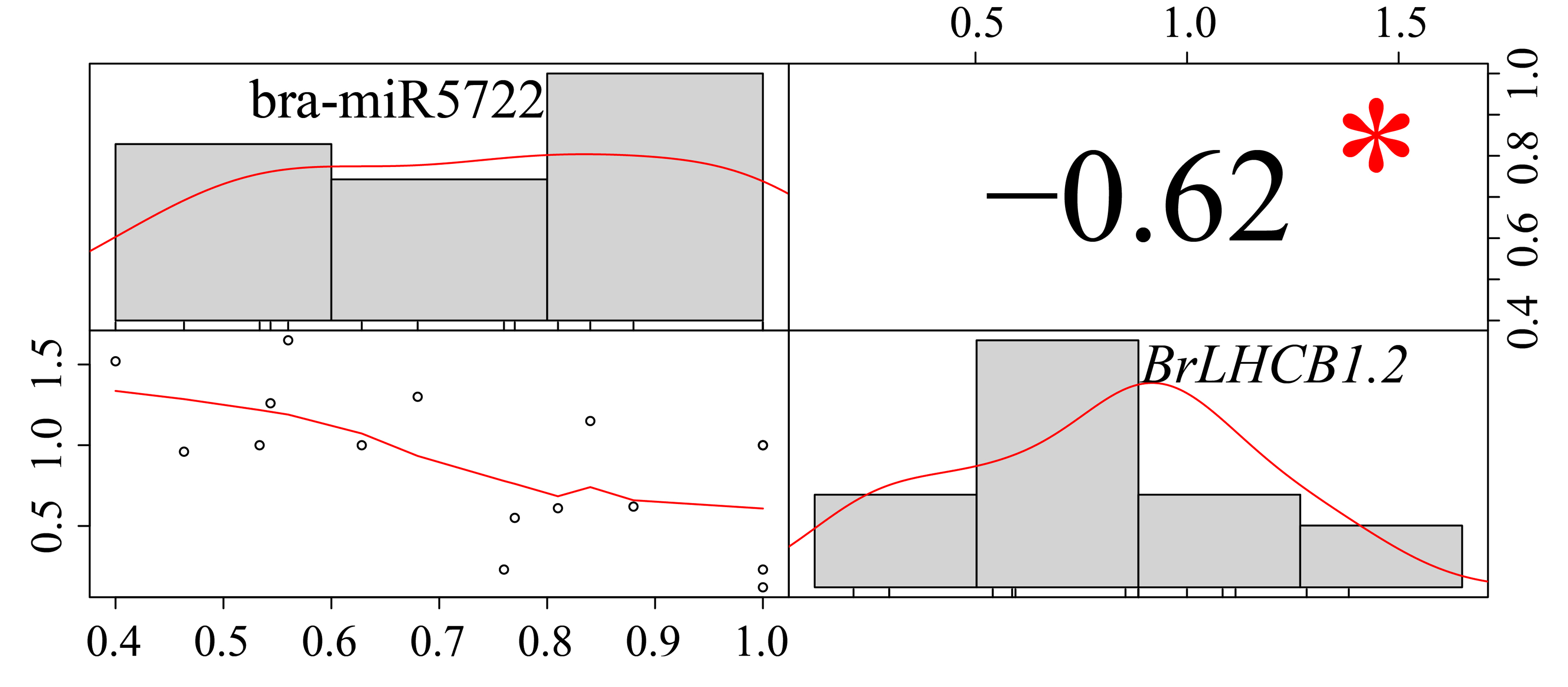

Supplement: Supplementary file 1 — Supplementary Figure S7. Correlation between bra-miR5722 and BrLHCB1.2 [file 41438_2021_474_MOESM1_ESM.jpg]

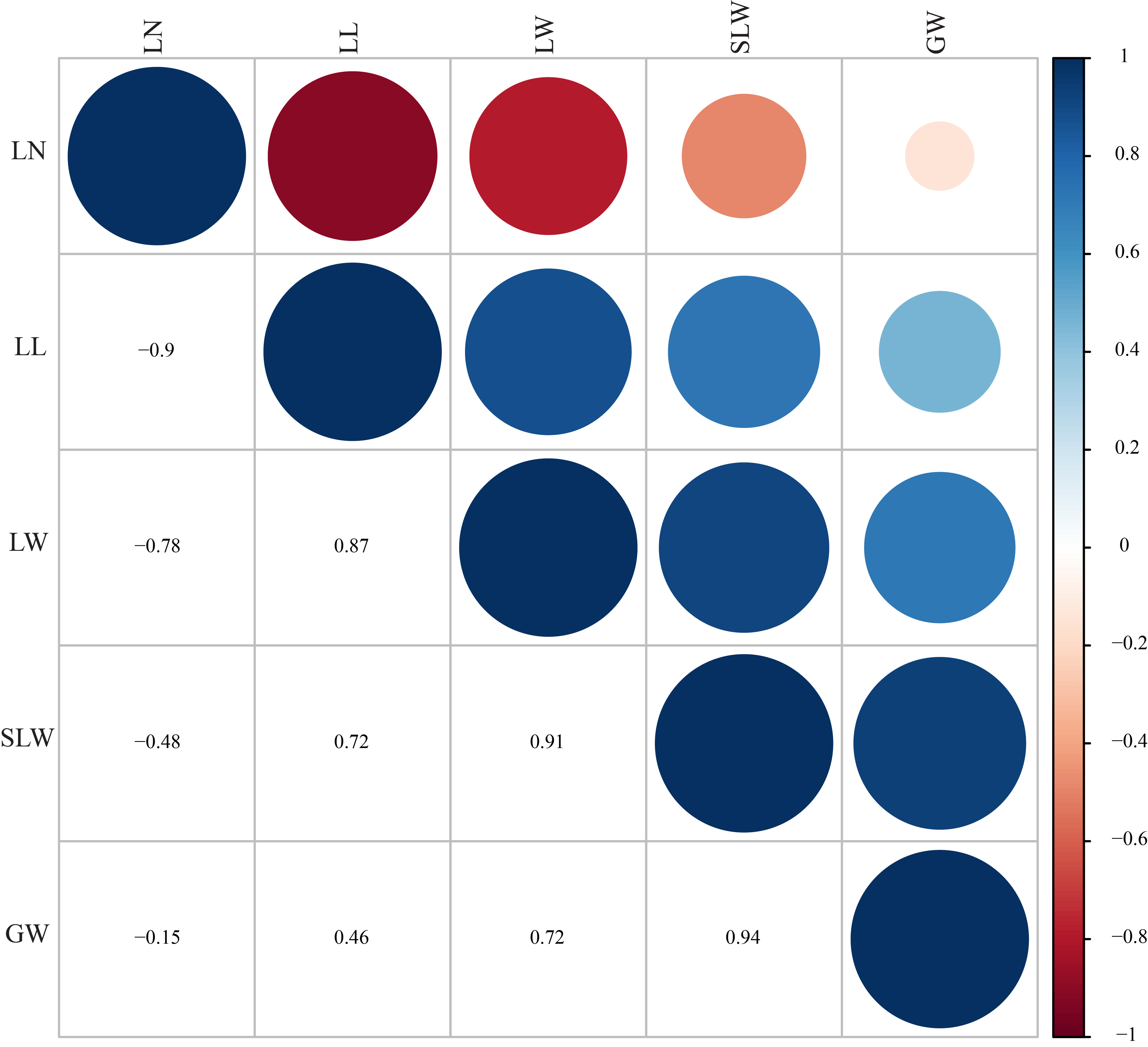

Supplement: Supplementary file 2 — Supplementary Figure S1. Correlation between all traits in the F1 hybrid and its parental inbred lines [file 41438_2021_474_MOESM2_ESM.jpg]

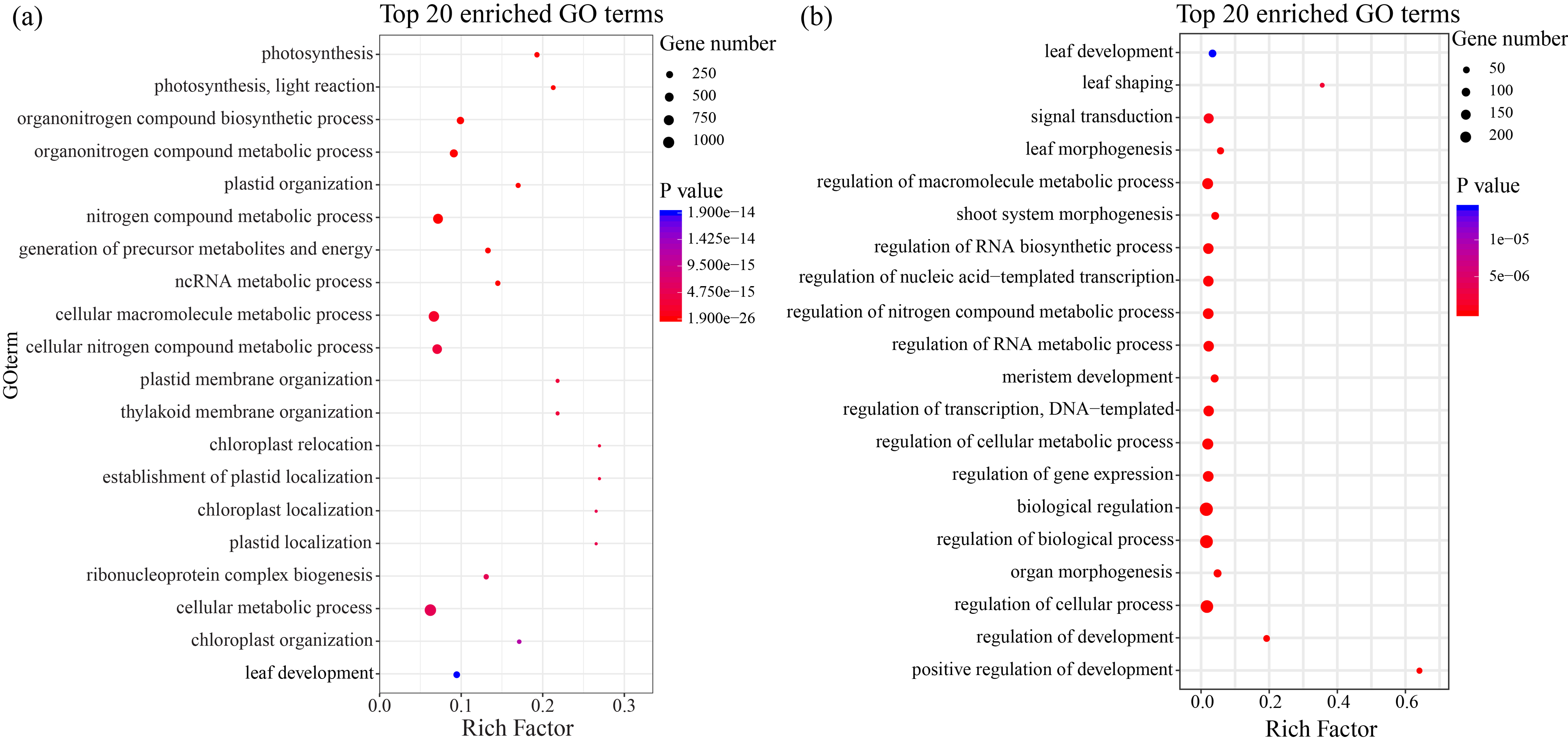

Supplement: Supplementary file 3 — Supplementary Figure S2. GO enrichment analysis of the DEM (a) and MVP-DEM (b) target genes [file 41438_2021_474_MOESM3_ESM.jpg]

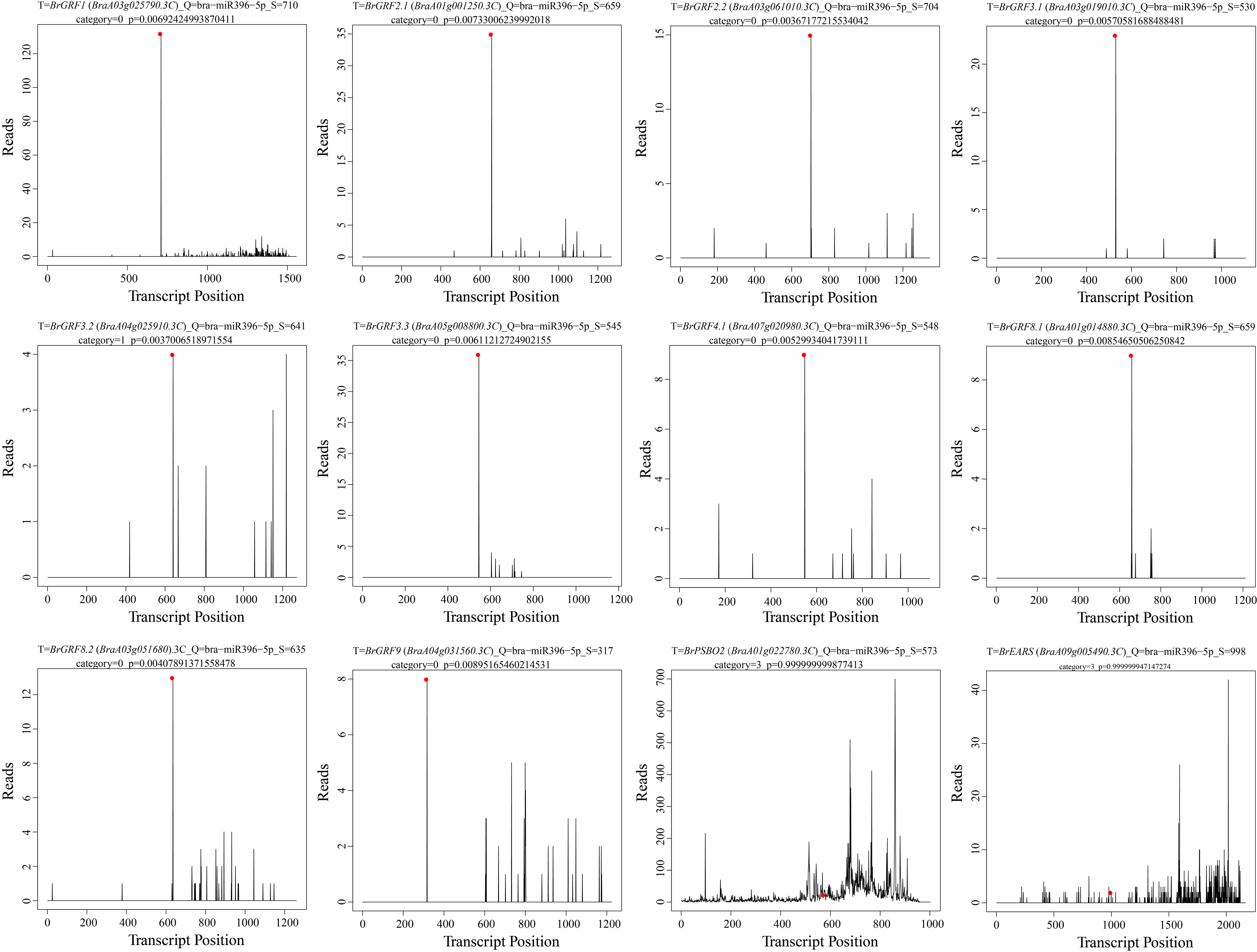

Supplement: Supplementary file 4 — Supplementary Figure S3. Target (T) plots of miRNAs validated by degradome sequencing [file 41438_2021_474_MOESM4_ESM.jpg]

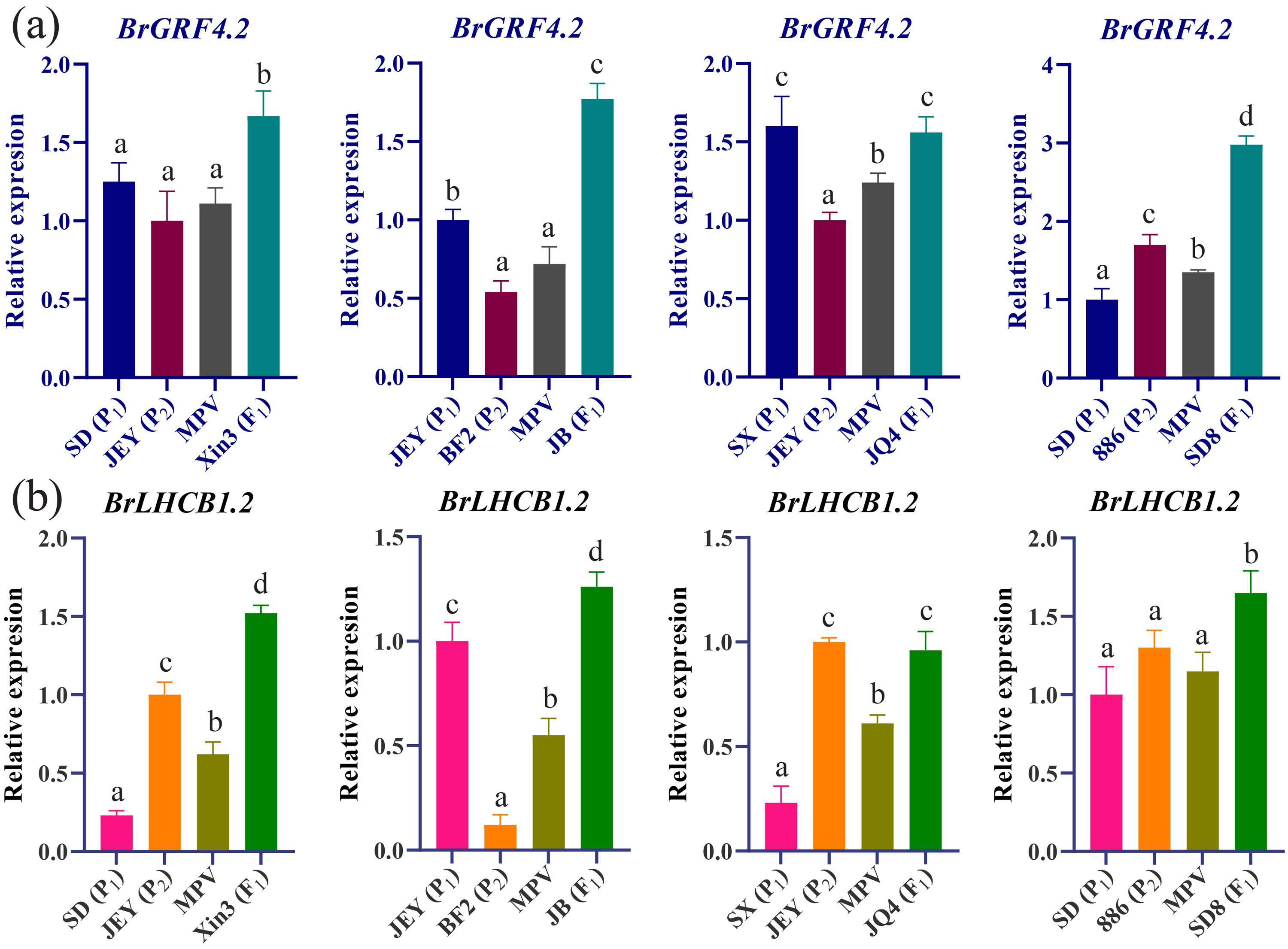

Supplement: Supplementary file 5 — Supplementary Figure S4. The expression of miRNAs’ targets in four Chinese cabbage F1 hybrids, their respective parental inbred lines, and the MPV [file 41438_2021_474_MOESM5_ESM.jpg]

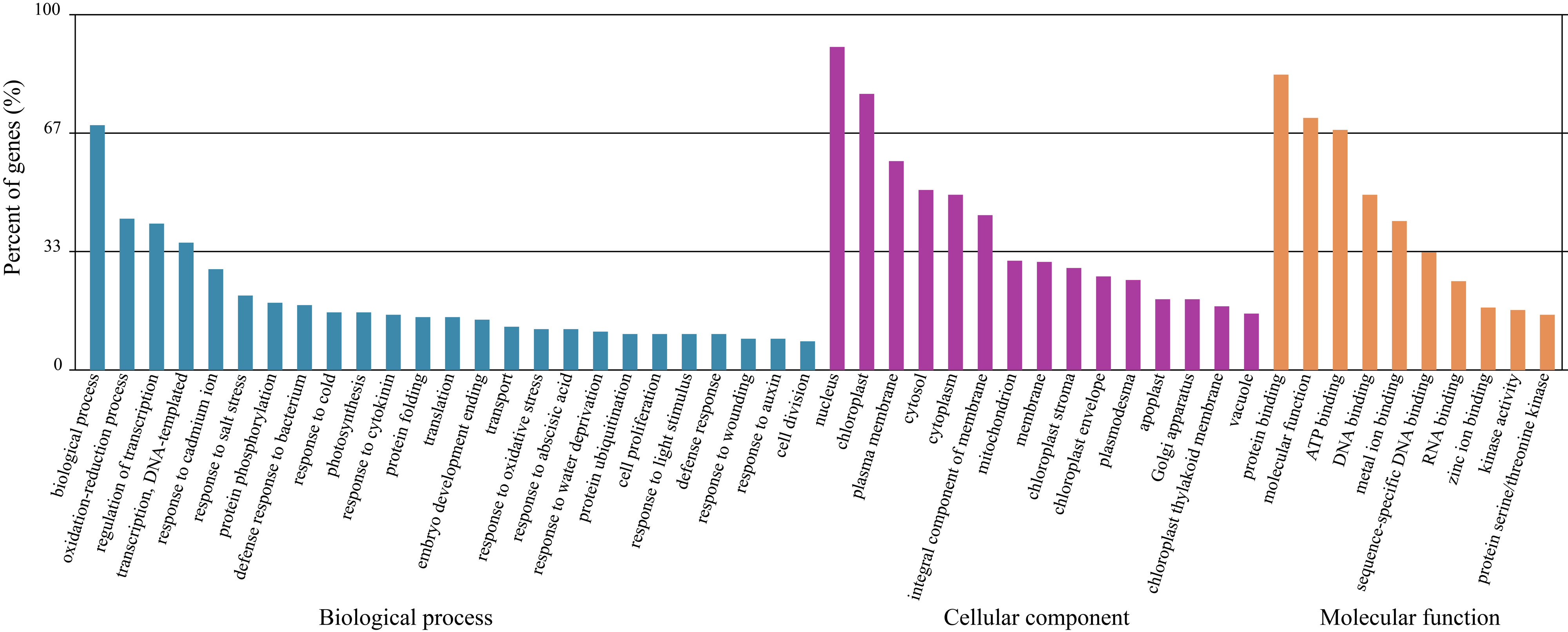

Supplement: Supplementary file 6 — Supplementary Figure S5. Functional annotation of enriched GO terms in the three main categories ‘biological process’, ‘cellular component’, and ‘molecular function’ for MPV-DEGs in Chinese cabbage [file 41438_2021_474_MOESM6_ESM.jpg]

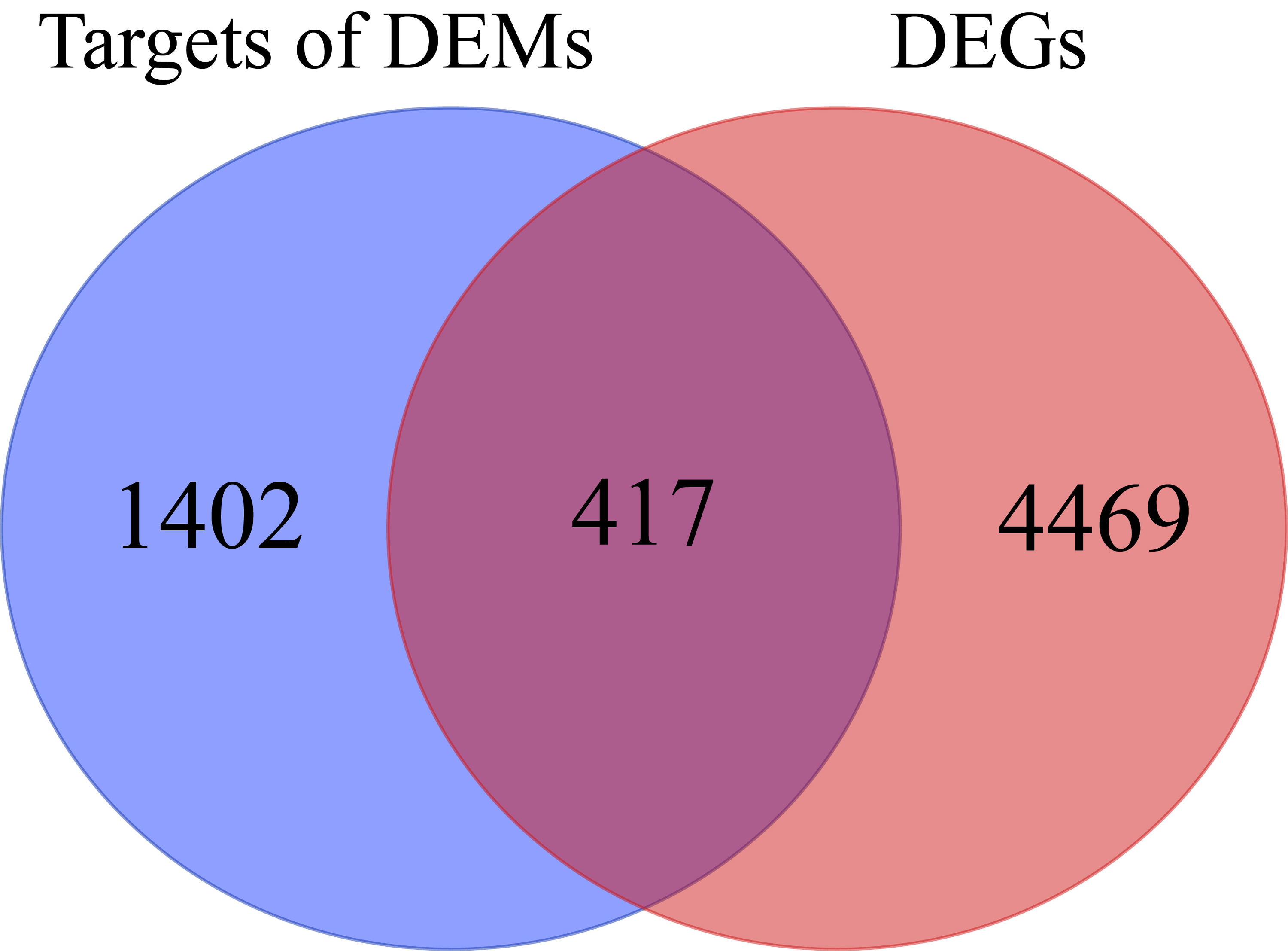

Supplement: Supplementary file 7 — Supplementary Figure S6. Venn diagram representing the number of DEM’s targets and DEGs [file 41438_2021_474_MOESM7_ESM.jpg]
